# Putting desire on a budget: dopamine and energy expenditure, reconciling reward and resources

**DOI:** 10.3389/fnint.2012.00049

**Published:** 2012-07-20

**Authors:** Jeff A. Beeler, Cristianne R. M. Frazier, Xiaoxi Zhuang

**Affiliations:** ^1^Department of Neurobiology, The University of ChicagoChicago, IL, USA; ^2^Committee on Neurobiology, The University of ChicagoChicago, IL, USA

**Keywords:** reward, energy management, dopamine, basal ganglia, incentive-salience, cost sensitivity, effort, explore-exploit

## Abstract

Accumulating evidence indicates integration of dopamine function with metabolic signals, highlighting a potential role for dopamine in energy balance, frequently construed as modulating reward in response to homeostatic state. Though its precise role remains controversial, the reward perspective of dopamine has dominated investigation of motivational disorders, including obesity. In the hypothesis outlined here, we suggest instead that the primary role of dopamine in behavior is to modulate activity to adapt behavioral energy expenditure to the prevailing environmental energy conditions, with the role of dopamine in reward and motivated behaviors derived from its primary role in energy balance. Dopamine has long been known to modulate activity, exemplified by psychostimulants that act via dopamine. More recently, there has been nascent investigation into the role of dopamine in modulating voluntary activity, with some investigators suggesting that dopamine may serve as a final common pathway that couples energy sensing to regulated voluntary energy expenditure. We suggest that interposed between input from both the internal and external world, dopamine modulates behavioral energy expenditure along two axes: a conserve-expend axis that regulates generalized activity and an explore-exploit axes that regulates the degree to which reward value biases the distribution of activity. In this view, increased dopamine does not promote consumption of tasty food. Instead increased dopamine promotes energy expenditure and exploration while decreased dopamine favors energy conservation and exploitation. This hypothesis provides a mechanistic interpretation to an apparent paradox: the well-established role of dopamine in food seeking and the findings that low dopaminergic functions are associated with obesity. Our hypothesis provides an alternative perspective on the role of dopamine in obesity and reinterprets the “reward deficiency hypothesis” as a perceived energy deficit. We propose that dopamine, by facilitating energy expenditure, should be protective against obesity. We suggest the apparent failure of this protective mechanism in Western societies with high prevalence of obesity arises as a consequence of sedentary lifestyles that thwart energy expenditure.

## Introduction

The idea that the primary function of dopamine is to mediate reward is pervasive. Although controversies abound over precisely *how* dopamine may contribute to reward—or even if it does (Cannon and Palmiter, 2003; Wise, 2004; Berridge, 2007; Goto et al., 2007; Robbins and Roberts, 2007; Salamone, 2007; Schultz, 2007; Redgrave et al., 2008), reward as an organizing metaphor for dopamine function is so ubiquitous as to often be treated as fact, a trend especially pronounced within the obesity and feeding literature where midbrain dopamine is effectively equated with reward (e.g., Kenny, 2010; Volkow et al., 2010; Avena and Bocarsly, 2011; Berthoud et al., 2011). However, decades of research have indisputably documented a clear role for dopamine in modulating activity, best illustrated by the psychostimulant properties of drugs that increase dopamine signaling. Salamone and colleagues have long argued that the primary effect of dopamine is to regulate effortful activity, allowing an animal to overcome response costs associated with pursuing valuable stimuli (Salamone, 2009, 2011). More recently, genetic studies exploring potential genes that regulate voluntary activity have pointed to dopamine related genes with some authors suggesting that dopamine may represent a “final common pathway” in controlling voluntary activity (Leamy et al., 2008; Kelly et al., 2010; Knab and Lightfoot, 2010; Mathes et al., 2010; Garland et al., 2011). Despite compelling and substantial data suggesting that dopamine plays a key role in energy expenditure, this view of dopamine is overshadowed by the reward perspective. For example, in many papers discussing dopamine and obesity (Geiger et al., 2009; Berridge et al., 2010; Kenny, 2010; Berthoud et al., 2011), dopamine's role in energy expenditure is not even considered, despite the fact that energy expenditure represents conceptually half of the energy balance equation.

To date, no compelling framework has integrated these two distinct domains of dopamine effects and putative function, the widely recognized reward function and the less prominent but equally demonstrable effects of dopamine on activity and energy expenditure. Apparent dopaminergic effects on activity are often framed as a consequence of reward processes. For example, the role of dopamine in modulating voluntary wheel running in rodents has been proposed to arise from dopaminergic modulation of the reward and reinforcement associated with wheel running (Garland et al., 2011; Roberts et al., 2011; Yang et al., 2012). Here we develop a hypothesis in which the primary function of dopamine is to regulate energy expenditure. Specifically, we argue that dopamine serves as an interface between the internal and external environments matching behavioral energy expenditure to the prevailing, environmental energy economy. We propose that dopamine regulates energy expenditure along two dimensions: (1) how *much* energy to expend (conserve-expend axis) and (2) how to *distribute* or allocate energy to different activities (an explore-exploit axis, elaborated below). In this view, dopamine's reward related effects arise secondary to and in the service of adaptively managing energy expenditure. We are profoundly indebted to Salamone's elegant work and dogged focus on the role of dopamine in regulating effort and his persistent criticism of the reward hypothesis of dopamine. The present hypothesis represents an integration and expansion of his fundamental insights into a broader hypothesis in which dopamine adaptively regulates both effort and reward—scaling the impact of prior reward history on current behavior—with respect to the availability of energy in the environment.

## Investigating dopamine in an adaptive, semi-naturalistic context

Below, we will first review recent studies from our laboratory that draw into question the primacy of reward in the dopaminergic modulation of behavior and highlight its role in energy expenditure. Subsequently, we will elaborate an energy economics hypothesis of dopamine function, reviewing relevant literature. We will conclude with a consideration of the present hypothesis in investigating the role of dopamine in obesity. The term “reward” is unfortunately abused within the literature, as has been pointed out by others (Cannon, 2004; Salamone et al., 2005; Salamone, 2006; Yin et al., 2008). In particular, the word is used imprecisely and ambiguously to capture different concepts, including affective responses (such as liking something), reinforcement (an outcome that increases the likelihood of the preceding behavior being repeated), stimuli that meet an appetitive need (e.g., food) and so on. In the first part of this review, we (mis-) use the term broadly, much like it is misused in the literature, as an umbrella term to lump together and capture themes that pervade the literature, despite various theoretical differences between different ideas. Subsequently, we will define reward more precisely as we develop our hypothesis.

### Elevated dopamine: decreased coupling to reward

Implicit in reward perspectives of dopamine is the idea that *dopamine enhances the impact of reward on behavior*. Empirically, this is supported by innumerable studies that show that increasing dopamine increases the effort an animal exerts toward reward while decreasing dopamine diminishes effort (Wise et al., 1978; Taylor and Robbins, 1986; Aberman et al., 1998; Peciña et al., 2003; Kelley, 2004; Cagniard et al., 2006a,b; Phillips et al., 2007; Salamone, 2009, 2011). These cumulative data have led many investigators to conclude that either the reward itself or incentive associated with reward related stimuli are enhanced by dopamine. Alternatively, the sensitivity to costs associated with obtaining the reward may be diminished by increased dopamine (Phillips et al., 2007; Salamone, 2011). What is often inferred from these studies, even if their authors adamantly oppose such interpretations (e.g., Salamone), is that dopamine modulates the relationship between reward and behavior in such a way that dopamine increases the degree to which reward biases behavioral choice. For example, Salamone argues that dopamine does not modulate reward at all; he shows that dopamine facilitates *effort*. Thus, the animals' pursuit of reward is less impeded by response cost. Many have interpreted this as increasing the impact of reward on behavioral choice not by changing reward itself but by altering a factor—response cost—that normally constrains reward pursuit.

To examine how increased dopamine alters adaptation to a semi-naturalistic environment, we asked whether or not the repeatedly observed increase in motivation for reward would result in diminished behavioral flexibility (Beeler et al., 2010). To test this, we used a home cage paradigm where the mice lived in operant equipped home cages and all of their food was obtained through lever pressing, 24/7. No food restriction was employed and mice were allowed to entirely self-regulate their consumption. Two levers yielded food where one was always “cheap” and required a low number of presses for a pellet (FR20) while the other was always “expensive” and required a greater number of presses that incremented as the experiment progressed (FR40–FR200). Which lever was which, however, switched randomly every 20–40 min. Thus, to obtain the greatest return for effort expended, the mice had to monitor on-going reward feedback and periodically switch levers to obtain the lowest cost pellets. We tested wild-type C57BL/6 (control) and dopamine transporter knock-down (DATkd) mice that have elevated extracellular dopamine and increased tonic dopamine firing activity (Zhuang et al., 2001; Cagniard et al., 2006b).

We found that the mice with elevated dopamine (DATkd) pressed significantly more on the high cost lever than wild-type mice, consistent with prior literature that shows dopamine enhances effort toward reward. However, in this instance the increased effort did not increase reward, merely the amount of effort expended toward that reward. Detailed analysis of the data show that the DATkd were not insensitive to or unaware of switches between the levers as their peri-switch behavior was essentially identical. The difference arose during the stable periods between lever switches where the DATkd mice distributed their effort equally to both levers while the wild-type mice preferentially pressed the cheap lever. To better understand the strategy underlying the DATkd behavior, we fit the data to a temporal difference learning (TD) model (Sutton and Barto, 1998). In these models, there are two key parameters: a learning rate that controls the rate at which new reward information is incorporated into (and fades from) the value of pressing the lever and an “inverse temperature” that controls the degree to which that value biases behavioral choice. The latter parameter is often termed the explore-exploit parameter as a greater bias result in exploitation of learning while reduced bias permits greater exploration (Sutton and Barto, 1998; Daw et al., 2006). We found that there were no differences between the genotypes with respect to learning rate, consistent with the lack of learning differences observed around switches, but that the DATkd exhibited a reduced inverse temperature. That is, there was a *reduced* coupling between reward history and their behavioral choices. At first glance this appears paradoxical. Although the DATkd mice worked harder to obtain reward, consistent with decades of literature, this does not seem to arise as a consequence of reward exerting a greater control over their behavior. On the contrary, there was diminished coupling between reward and behavioral choice. Rather than reward having a greater biasing effect on their behavior, it had *less*. Increased dopamine, under these conditions, resulted in decreased rather than increased exploitation. Interestingly, Salamone et al. (Salamone et al., 2001) have demonstrated that rats with nucleus accumbens dopamine depletions are *more* dependent on recent reward to overcome response cost, suggesting the converse under reduced dopamine, an *increased* coupling between reward history and choice.

### Elevated dopamine: modulation of effort without increased consumption

Another idea implicit in the reward perspective of dopamine is that dopamine, by increasing the degree to which reward biases behavior, regulates the degree to which an animal will pursue reward; that is, that *dopamine mediates “wanting”*: more dopamine, more wanting, more pursuit (Robinson and Berridge, 1993; Leyton et al., 2002; Volkow et al., 2002; Tindell et al., 2005; Berridge et al., 2010). This effect of dopamine is central to many theories of addiction (Robinson and Berridge, 2001; Koob and Volkow, 2010) and, more recently, theories of dopamine and obesity (Volkow and Wise, 2005; Finlayson et al., 2007; Zheng et al., 2009; Berridge et al., 2010; Volkow et al., 2010; Avena and Bocarsly, 2011; Berthoud et al., 2011). In another recent homecage study (Beeler et al., 2012a), we asked whether or not under different environmental conditions this increased “wanting” could be adaptive. To test this, we again housed mice in home cages equipped with operant levers where they earned all their food through lever pressing, again without explicit food restriction. In this paradigm, only one lever yielded food and the work demand for that lever incremented every 3 days throughout the experiments, starting at FR5 and ending at FR250. This yields a demand curve that shows the degree to which mice adjust their daily consumption to the current cost of pellets. Assuming that dopamine increases the value of reward and/or decreases sensitivity to cost, we would expect that the DATkd mice with elevated dopamine would fare better in this paradigm and continue pressing more at higher costs than wild-type mice. Although the DATkd did show mildly increased pressing at higher costs, overall they exhibited the same adjustment to escalating costs as wild-type mice, with no difference observed in body weight changes or survival within the experiment. Moreover, when the data were fit to a model of demand elasticity (Hursh and Silberberg, 2008), there was no difference between the genotypes in elasticity. So where did the dopamine effect on effort and reward go?

Analysis of individual meal data (i.e., number, duration, and size of meals) indicates a large genotype effect where the DATkd mice ate *larger* but *fewer* meals. That is, although dopamine did not significantly change their overall consumption, it did change their meal patterning—the way in which they temporally distributed their effort and consumption. These data suggest that escalating costs induced a condition of scarcity that engaged homeostatic conservation mechanisms in both the wild-type and DATkd. To avoid this artificial condition of scarcity, we conducted a home cage progressive ratio study where the escalating costs occur within each meal or bout of pressing as the cost of each subsequent pellet increases by 2. After a 30 min cessation of all pressing, the ratio reset. In this way, mice could shift their effort toward larger, more costly meals, or smaller, cheaper, and more frequent meals without sacrificing overall consumption. In this study, we observed no significant body weight changes between groups and no significant difference in overall consumption. However, the DATkd mice, again, ate larger meals and exhibited a higher breakpoint within individual bouts, consistent with previous literature showing that elevated dopamine increases breakpoint in the progressive ratio paradigm. However, as above, this greater effort was offset by less frequent meals such that overall consumption was not different. From these studies, we draw two important conclusions. First, the effects of dopamine on pursuit of food, at least in this paradigm, appear to remain under homeostatic control. Second, dopamine does not appear to alter “wanting” or overall pursuit of food in a global sense but appears to modulate effort expended within temporally local episodes of goal pursuit. In short, dopamine appears to have affected the way energy and effort is *distributed* rather than increasing appetitive motivation *per se*.

We observe here that increased dopamine does not make demand more inelastic; that is, *overall* hyperdopaminergic mice adapt their consumption to response costs similarly to wild-type. On the surface this appears contradictory to many studies that suggest that stimulating or impeding dopamine transmission can enhance or diminish effort-based responding, respectively (e.g., Aberman and Salamone, 1999; Bardgett et al., 2009; Salamone et al., 2009b), presumably influencing elasticity in response to costs. However, we observe the same phenomenon observed in those studies: dopamine facilitates greater effort during a *bout* of food pursuit, evidenced here by larger meals and higher breakpoints. However, we also observe what session based studies cannot—that these differences in effort, from which we might infer changes in elasticity, are not necessarily accompanied by changes in overall consumption and demand. Those larger meals are compensated by fewer meals, resulting in an overall similar elasticity in response to escalating costs. That elevated dopamine did not produce inelasticity in these studies does not mean that dopamine never modulates elasticity, only that the relationship between dopamine, effort and demand may be more complex than previously appreciated.

### Elevated dopamine: does not enhance hedonic value or shift behavioral choice

Another idea implicit in the dopamine and reward hypothesis is that *dopamine increases pursuit of* preferred *foods* (Salamone et al., 1991; Cousins et al., 1993; Salamone, 1994; Lowe and Levine, 2005; Zheng et al., 2009; Berridge et al., 2010; Kenny, 2010; Volkow et al., 2010), where “preferred” is typically defined as palatable, hedonically rewarding foods: things that taste good. From an incentive-salience perspective, dopamine enhances the greater incentive associated with preferred foods. Arguing against this, Salamone et al. (Salamone et al., 1991; Salamone, 1994) have demonstrated that under free-feeding conditions, preference is not altered by changes in dopamine function; that is, dopamine does not increase incentive or alter food preference when no (or low) work requirement is present. In his studies, however, when obtaining a preferred food *is* associated with a response cost, dopamine increases the effort an animal will exert, thus altering the animal's *behavioral choice* in favor of greater pursuit of a preferred food (Salamone, 1994; Salamone et al., 1994), which is often taken to suggest that increased dopamine would increase pursuit of preferred food in a naturalistic foraging environment by reducing sensitivity to associated costs.

In a recent series of studies (Beeler et al., 2012b), we examined the relative contribution of nutritional and hedonic, or taste, value to consumption, preference, and reinforcement and asked how elevated dopamine may alter these. To test for taste value alone, we used calorie-free sweeteners (both sucralose and saccharin). To test for nutritional value alone, we used trpm5 knock-out mice that lack the sweet taste receptor and do not taste sweet (Damak et al., 2006; de Araujo et al., 2008), allowing us to assess the impact of nutrition alone. In both cases, we used mice with and without a knock-down in the dopamine transporter to test the effects of elevated dopamine. The literature suggests that more hedonically rewarding foods would be more affected by increased dopamine. From this, we might predict that elevated dopamine would preferentially affect hedonic over nutritional reward. We found, first, that although both hedonic and nutritional value induced increased consumption and preference, hedonic value dissociated from nutritional value was a poor reinforcer. That is, mice would consume calorie-free sweet solutions and prefer it to water, but sweet taste in the absence of caloric value lacked the capacity to induce conditioning in the two bottle conditioning test. Moreover, in a progressive ratio test, sucrose induced increased responding across sessions. Calorie-free sweeteners, in contrast, induced much less responding that actually declined across sessions, resembling so-called “extinction mimicry” (Wise et al., 1978). Berridge and Robinson (Robinson and Berridge, 1993) have famously described a dissociation between “wanting” and “liking” in addiction where addicts develop “wanting” for drugs without “liking”; that is, the incentive driving compulsive drug seeking is independent of its hedonic consequences. These data suggest a complementary dissociation of “liking” without “wanting” where one can experience a positive hedonic response without developing associative incentives that drive compulsive seeking of that experience in the future (Beeler et al., 2012b).

Contrary to expectation, elevated dopamine did not significantly change motivation for hedonic, sweet taste alone but did increase effort for combined taste/nutrition as well as nutrition alone. Previous studies of dopamine release have shown that dopamine is released in response to taste alone (e.g., using intra-oral cannulation to stimulate taste without postingestive effects) (Mark et al., 1991; Hajnal et al., 2004; Norgren et al., 2006; de Araujo et al., 2008; Wheeler et al., 2011). So why did we observe reduced reinforcement to sweet taste in the absence of nutrition? We conducted a voltammetry study in which the rats were pre-exposed to both sucrose and saccharin pellets, each with an identifying flavor, to provide them the opportunity to discriminate and learn about the relative nutritional value of each (Beeler et al., 2012b). We then measured evoked dopamine release in response to either sucrose or saccharin. The rats retrieved and consumed both types of pellets equally; however, when we measured evoked dopamine release, the response to saccharin was greatly attenuated compared to sucrose. In a follow-up study, the same attenuation of evoked dopamine was observed in response to cues predicting either sucrose or saccharin (McCutcheon et al., 2012). The attenuated dopamine response to calorie-free taste alone is consistent with the reduced responding and apparent extinction mimicry observed in the behavioral studies with the mice. In short, these studies show that increased dopamine, though it increases effort and alters the distribution of energy expenditure (i.e., meal patterns), did not alter consumption or preference and did not augment “wanting” of hedonically valued foods in the absence of nutrition.

In the conventional concurrent choice task (Salamone, 1994) an animal has a choice between lever-pressing for a preferred food or eating freely available standard chow during one hour sessions. Salamone and colleagues have shown that dopamine increases the ratio of preferred food to standard chow consumed; that is, dopamine shifts behavioral choices to favor the more costly but preferred option. Many infer from this work that dopamine will increase pursuit of preferred foods. We tested this inference by conducting a homecage progressive ratio concurrent choice experiments where mice could lever press for a preferred food (PR2), either calorie-free sweeteners or sucrose, or eat freely available chow. In this semi-naturalistic paradigm, increased dopamine, as reported in the demand and homecage progressive ratio studies above, shifted the distribution of effort toward greater energy expenditure (i.e., longer bouts of pressing, greater breakpoint, but fewer overall bouts). Despite greater effort, however, elevated dopamine did not alter their behavioral choice as reflected in the ratio of preferred food to standard chow. Salamone has argued that enhanced effort toward a preferred food observed in the concurrent choice paradigm reflects alterations in sensitivity to response cost and not altered preference (Salamone et al., 2007). These data confirm and extend this argument by falsifying the inference that the increased effort observed in the concurrent choice paradigm will increase reward pursuit. In a semi-naturalistic environment, we observe the same enhanced effort toward a preferred food observed by Salamone, but this does not shift consumption, preference or behavioral choice but reflects different energy expenditure strategies.

## Integrating reward and behavioral energy management

These findings are difficult to explain solely in terms of current theories of dopamine and reward. (1) Rather than increasing the impact of reward on behavior, we observe a reduced coupling between reward history and choice, suggesting dopamine induces greater exploration; that is, *less* biasing of choice by reward. (2) Rather than over-riding homeostatic mechanisms and promoting excess consumption, dopamine appears to work within the constraints of homeostatic regulation, altering the *distribution of effort* in pursuit of food without changing overall consumption; that is, dopamine induces greater vigor but not greater “wanting.” (3) Rather than shifting effort, consumption, and behavioral choice to more preferred foods, dopamine again increases vigor without altering consumption, preference or choice; that is, the apparent decreased sensitivity to costs does not shift appetitive goals. The studies described all point to a common theme: that dopamine is modulating behavioral energy *expenditure*. Dopamine modulation of effort and expenditure has been viewed as either a non-specific effect—“generalized activity”—and/or as a reallocation of effort that overcomes response-costs associated with pursuing goals (Salamone et al., 2007). In the remainder of the paper, we will attempt to integrate the reward and activity modulating aspects of dopamine. To do so, we will develop an alternative perspective: the effects of dopamine on reward arise secondary to and in the service of regulating behavioral energy expenditure, placing the reward system within a larger context of reconciling energy expenditure with available resources.

### Dopamine: a behavioral energy management system

**Figure 1:**
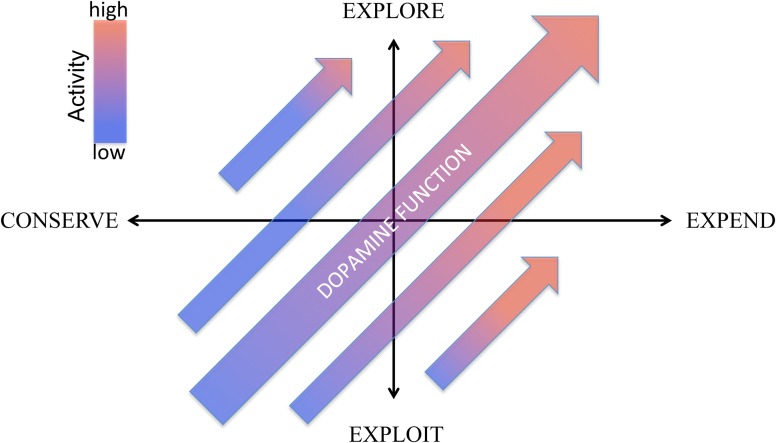
**Two axes conceptual framework for regulation of behavioral energy expenditure by dopamine.** The horizontal axes represents dopamine's role in regulating generalized activity levels along a continuum from low activity (conserve) to high activity (expend). The vertical axes represent the role of dopamine in regulating the balance between exploration and exploitation by modulating the degree to which reward information biases the distribution of behavioral activity. “Dopamine function” is construed broadly here and may include not only extracellular concentrations of dopamine in target regions, activity of dopamine neurons (i.e., rate of tonic activity, prevalence of bursting) but also parameters such as relative expression of different receptors (e.g., D1 and D2), expression and activity of the doapmine transporter (DAT) as well as properties of vesicular release, including size of readily releaseable pool and vesicle size. As a general conceptual principle, we associate reduced dopamine function with conservation and exploitation (lower left quadrant) and increased dopamine function with expenditure and exploration (upper right quadrant), as reflected by the larger arrow. However, alterations of different aspects of the dopamine system (for example, shifting the relative expression of D1 and D2 receptors) may shift this relationship, generating behavior described by the other quadrants, such as high expenditure coupled with a greater exploitation of reward information (lower right quadrant).

Energy and its use is the final bottom line for adaptation. All organismal needs and functions, from thermoregulation to reproduction to procurement of energy itself, require energy. Maintaining an adequate supply might be thought of as an evolutionary prime directive. Much focus has been given to systems that regulate the pursuit, consumption and storage of energy, but much less attention to systems that control its expenditure. Yet, aside from being an “equal partner” in determining energy balance (i.e., consumption—expenditure = net balance), achieving an optimal distribution of energy to different activities is critical to adaptation. That is, what an animal does with its available energy is arguably as important as acquiring energy. How to best direct energy expenditure, however, is contingent upon environmental conditions. In an energy rich environment, exploration, exercise, and energy expenditure is adaptive. In an energy poor environment, exploitation of prior experience and energy conservation—getting the most bang for one's proverbial energy buck—is essential. In this view, effective energy management entails deciding (1) how much energy do I have to expend and (2) how carefully, or selectively, do I need to deploy it. We characterize these two questions as two axes of energy management: expend vs. conserve and explore vs. exploit, respectively (Figure 1).

In the current hypothesis, we associate these two axes of energy expenditure with two well-documented dopamine functions. First, we link to dopamine's role in regulating generalized activity to the expend-conserve axes. Second, we suggest that dopamine's role in reward is not to modulate reward incentive and appetitive pursuit *per se*, but to use *reward information* to regulate the *distribution* of energy to different activities, mediating the explore-exploit axes; that is, how selectively should energy be deployed? We frame this second aspect of dopamine within TD learning models of dopamine and reward where dopamine mediates both (1) learning *about* reward—that is, assigning value to stimuli that directs energy expenditure and (2) the degree to which that reward information biases behavioral choice, construing the second axis, explore-exploit, as *energetic thrift*: how carefully do I need to exploit my available resources?

By emphasizing the management of energy expenditure rather than reward modulation as a primary function of dopamine, many otherwise hard to reconcile observations can cohere into a central organizing metaphor for understanding the role of dopamine in behavior. In the following sections, we briefly detail the core elements of this hypothesis in the context of current ideas and literature about dopamine.

It should be noted that the dopamine system is complex and multi-faceted. Aside from extracellular dopamine concentrations and rates of tonic and phasic dopamine cell firing, “dopamine function” can include modulation of release at its targets, modulation of synthesis, vesicular packaging and readily releasable pools, changes in receptor expression and function and alterations in dopamine transporter and reuptake. As a necessary simplification for the purposes of exposition, we will speak of “high” and “low” dopamine function, a not uncommon practice in the literature. The complexities this obscures represent potential mechanisms by which the dopamine system can exert more nuanced, flexible and sophisticated regulation of its function(s). In Figure 1, it is these complexities that allow us to consider dopamine not as a single line from high to low (as shown in the middle) but as a more complex function that could potentially range across the two-dimensional space portrayed. Understanding these complexities, however, depends upon an interpretative framework around which to organize more detailed information. Here we focus on articulating a *skeletal* alternative framework and do not attempt to assimilate everything known about dopamine nor detail every mechanism potentially involved in dopamine signaling, an intractable challenge for a single paper.

### Dopamine: mediating between the internal and external worlds

The dopamine system is interposed between two worlds of stimuli: the external and internal. On one hand, dopamine modulates an organism's response to environmental stimuli. In the reinforcement learning perspective of dopamine and reward (Montague et al., 1996; Schultz et al., 1997), dopamine critically mediates learning about the value of stimuli (state) and which responses (actions) are optimal (Reynolds et al., 2001; Schultz, 2002; McClure et al., 2003; Daw and Doya, 2006; Day and Carelli, 2007; Day et al., 2010; Flagel et al., 2010; Gan et al., 2010; Day et al., 2011). The incentive-salience perspective argues that dopamine scales the incentive value associated with environmental stimuli, altering the degree to which stimuli bias behavioral choice (Phillips et al., 2003; Berridge, 2004; Roitman et al., 2004; Cagniard et al., 2006b; Day et al., 2006; Cheer et al., 2007). In both cases, though mechanistically very different, dopamine is modulating the organism's response to environmental stimuli, the world outside.

More recent work has demonstrated complex interactions between the dopamine and homeostatic systems that monitor and signal information about the internal organismal milieu (Davis et al., 2010a; de Araujo et al., 2010; Figlewicz and Sipols, 2010; Opland et al., 2010; Vucetic and Reyes, 2010). Midbrain dopamine neurons express receptors for numerous circulating signals associated with homeostatic mechanisms, including leptin, ghrelin, orexin, and insulin (for extensive review, Figlewicz and Sipols, 2010). In addition to direct sensing of homeostatic signals, dopamine nuclei receive projections from various substrates associated with homeostatic control mechanisms, including hypothalamic projections (Opland et al., 2010). It is widely believed that these inputs modulate reward processes. For example, it is frequently proposed that circulating leptin decreases dopamine activity that in turn diminishes the reward value of food, consequently reducing appetitive behavior (Morton et al., 2009; Davis et al., 2010a; Figlewicz and Sipols, 2010; Opland et al., 2010; Vucetic and Reyes, 2010). The exact role of these homeostatic inputs remains controversial. The key point here is that the dopamine system receives substantial information about the internal milieu and homeostatic state of the organism, putting it in a position to exploit the organisms knowledge of its environment in accordance with internal needs and demands; that is, to modulate behavior to optimize the relationship between these two worlds, the inner and outer, organism and world. Of course, in a sense the entire brain has evolved to mediate between the inner and outer worlds, but the broad and diffuse projections of the dopamine system, together with the diverse inputs that converge upon it and its apparent role in modulating a wide array of behaviors and processes, from motivation to motor execution together with being highly conserved across species suggests it may, in fact, have evolved to play some fundamental, critical role in adaptation.

### Dopamine: putting desire on a budget

Few would argue with this notion that dopamine integrates internal and external information to adapt behavior to environmental conditions and optimally meet organismal needs. The difficult question is *how* does dopamine achieve this? That is, what is the primary *effector* mechanism by which dopamine adapts behavior? The prevailing view, almost hegemonic, is that dopamine modulates reward processes—regardless of whether it mediates learning about reward, the expression of incentive or some combination of both—and consequently shapes motivation: the goals and activities an organism pursues and the vigor with which these are pursued. Critically, the locus of modulation is *appetitive*: how much reward induces its pursuit.

Though much less discussed, dopamine also modulates activity levels. Consistent with this modulation of activity, Salamone and colleagues have long argued that dopamine can modulate both effort toward a goal (Salamone et al., 1997, 2005, 2009a) as well as generalized activity levels (Cousins et al., 1993; Correa et al., 2002), observations central to the current hypothesis.

We will argue that dopamine regulates energy expenditure to reconcile behavior with energy resources; that is, dopamine does not modulate desire, it puts it on an energy budget. In this view, energy availability, not reward, is the primary factor influencing dopaminergic regulation of behavior. In the following sections we will elaborate this energy management hypothesis of dopamine discussing first effector mechanisms by which dopamine regulates energy expenditure followed by a discussion of mechanisms by which the dopamine system regulates the distribution of energy using reward information. After elaborating the hypothesis, we will focus specifically on an alternative account of the role of dopamine in obesity.

## Axis I: conserve-expend

In the following sections, we will detail the role of dopamine in modulating energy expenditure along a continuum of conservation and expenditure. In the view developed, regulating energy expenditure—both generalized activity and effort toward particular goals—is fundamentally independent of reward and depends instead on available energy resources. Reward, we will argue, plays a distinct role in determining the *distribution or allocation* of that energy expenditure, represented by the explore-exploit axis and discussed in section “Axis II: Explore-exploit.”

### Dopamine and generalized activity: spend or save?

Elevated dopamine has been associated with increased activity for decades. Drugs that increase dopamine release, such as amphetamine, cocaine or dopamine reuptake inhibitors, increase generalized activity in humans and rodents (Kelly, 1975; Mogenson et al., 1980; Beninger, 1983; Ahlenius et al., 1987; Carlsson, 1993; Xu et al., 1994; Sedelis et al., 2000; Correa et al., 2002; David et al., 2005; Viggiano, 2008; Charntikov et al., 2011). Administration of D1 agonists or antagonists increase and decrease activity, respectively. D2 acting drugs act post-synaptically on medium spiny neurons in the inhibitory, indirect pathway; however, they also act pre-synaptically on dopamine and glutamate terminals and as autoreceptors on dopamine cell bodies. As a consequence, low doses of quinpirole, a D2 agonist, will depress activity presumably decreasing dopamine release through autoreceptor activation while high doses of quinpirole increase activity, presumably through activation of postsynaptic D2 receptors that decrease activity in the inhibitory, indirect pathway (Lomanowska et al., 2004). In rodents, some addictive drugs of abuse that are CNS depressants (e.g., morphine) increase activity, an effect believed to arise from increased dopamine release (Koek et al., 2012). Drugs that block dopamine reuptake increase activity (Billes and Cowley, 2008; Young et al., 2010) and DAT expression correlates with locomotor activity. Mice with reduced expression of the dopamine transporter, resulting in elevated tonic dopamine, are hyperactive (Cagniard et al., 2006a).

Though there is no doubt that dopamine modulates generalized activity, the mechanism by which it does so is poorly understood. In fact, there is no general framework for conceptualizing what, exactly, “generalized activity” or arousal is in the first place (Quinkert et al., 2011). In rodents, generalized activity is typically measured using either the open-field, running wheels or homecage activity monitors. The degree to which each reflects a general activity level is debated (Dishman, 2008; Viggiano, 2008; Hesse et al., 2010; Garland et al., 2011). The open-field, for example, can be viewed as a measure of general activity, exploratory behavior or “emotionality.” The running wheel, because running is highly reinforcing in rodents (Wagner, 2005; Brené et al., 2007; Greenwood et al., 2011), may be confounded with reward processes. Nonetheless, increasing dopamine increases activity on all three measures and conversely, decreasing dopamine decreases activity on all three measures (Ahlenius et al., 1987; Zhuang et al., 2001; Correa et al., 2002; Leng et al., 2004; Beeler et al., 2006, 2009; Dishman, 2008; Kitanaka et al., 2012).

Rather than viewing “generalized activity” as distinct from goal-oriented, reward-driven behavior, an alternative perspective would be that dopamine signals energy availability and induces energy expenditure, whether that be directed toward a rewarding, reinforced activity such as wheel running (or lever pressing), toward exploration such as in the open field or merely moving around doing more mouse-like stuff day to day in the home cage. As psychostimulant users for decades attest: dopamine is energizing. Thus, the effect of dopamine on generalized activity represents a fundamental effector in the regulation of energy expenditure by up- or down-regulating how much energy is expended in behavioral activity independent of how that activity may be directed: *independent of reward*.

### Dopamine and effort: how much can i afford?

The literature is replete with evidence that increased dopamine increases effort in pursuit of goals, canonically illustrated by increased performance in progressive ratio paradigms where after each reward earned, the cost of each subsequent reward increases (Hodos, 1961). Historically, the effort a subject makes in a progressive ratio test has been construed as a measure of reinforcer efficacy: that is, how hard I work is a measure of how valuable or motivating the reward is (Madden et al., 2007a,b). This is analogous to assessing the value of something according to the price someone is willing to pay.

However, breakpoint implicitly measures a cost-benefit determination (Salamone et al., 2009a). Though dopamine contributes to this on-going determination, it's role remains unclear (Salamone et al., 1997; Roesch et al., 2007; Day et al., 2010, 2011; Ostlund et al., 2010). On one hand, as argued in the incentive-salience theory, dopamine may enhance the incentive properties of stimuli associated with reward, essentially increasing the perceived *benefit* (Berridge, 2007; Gan et al., 2010). On the other hand, Salamone and colleagues argue that dopamine reduces sensitivity to costs, thus reducing the *cost* component (Salamone, 2011, see also Phillips et al., 2007). In both cases, the result is increased pursuit of reward arising from altered cost-benefit determination, observed as an elevated breakpoint. In the conventional progressive ratio paradigm, it is difficult to discern these two possibilities as the behavioral outcome would look the same: increased effort and responding. The home cage studies reviewed above, however, can distinguish between these two alternatives. If increased dopamine were increasing sensitivity to reward, inducing greater “wanting” and up-regulating appetitive motivation, then we would expect to see greater consumption and enhanced preference for more rewarding foods. We observed neither. Instead, we observed similar consumption, preference, and behavioral choices but a shift in foraging strategies toward greater energy expenditure. However, it would be incorrect to say that the DATkd mice are *insensitive* to costs; they adjust their consumption and effort in response to escalating cost just like the wild-type mice. Moreover, in the cheap-expensive lever switching paradigm, if the levers do *not* switch, the DATkd mice prefer the cheap lever identically to the wild-type. Rather, increased dopamine appears to shift foraging strategy toward greater energy expenditure.

In determining the optimal trade-off between cost and benefit, both factors are situationally contingent. On one hand, benefit is contingent upon need. A food pellet will be much more valuable and motivating to a hungry mouse than a sated one. The role that motivational state plays in determining reward and reinforcement has a long history in psychology and neuroscience (Berridge, 2004) and represents an active area of investigation (Dayan and Balleine, 2002; Balleine, 2005; Fontanini and Katz, 2009; Haase et al., 2009). Much less appreciated, however, is that the evaluation of cost may also be contingent. Specifically, the cost associated with any expenditure depends upon available resources. The cost of a $12.00 airport hot dog is not evaluated the same by a millionaire CEO and a poor graduate student. With rodents, similar resource contingencies may apply. For example, delay costs may be more significant if only a limited amount of time is available (such as in one hour operant sessions) for obtaining reward. Similarly, the cost associated with lever presses may depend upon the general availability of energy to a mouse. So if dopamine reduces cost sensitivity, there are two interpretations. In the first, cost sensitivity is decreased relative to benefit; that is, when cost and reward are compared, dopamine diminishes the cost factor in order to favor reward (functionally equivalent to increasing the incentive value). In the second, dopamine modulates cost-sensitivity *relative to available resources*. If energy is abundantly available, effort costs are discounted.

Thus, we argue that the dopamine regulation of effort is independent of reward value; that is, dopamine's effects on effort reflect a direct modulation of energy expenditure in relation to available energy resources rather than an indirect consequence of modulating a trade-off between cost and reward. As dopamine increases energy expenditure generally, as discussed above, so it increases energy expenditure—or vigor of goal pursuit—in specific activities, again, we argue, *independent of reward value*: if you have energy, use it.

## Axis II: explore-exploit

In the following sections we argue that reward serves a central function in the dopamine mediated management of energy: controlling the *distribution* of energy expenditure, which we represent with the explore-exploit axis (Figure 1). In this view, learned reward value(s) determines the relative utility of different activities; however, what is crucially captured in this axis is the *degree* to which these values (and the contrast between them) actually bias and shape behavioral choices. We integrate into this axis reinforcement learning and incentive-salience perspectives arguing that phasic dopamine mediates learning about and updating reward values, as widely believed, while *tonic* dopamine, as incentive views suggest, scales the impact of these values on behavioral choice, the expression of learned values. We reconceptualize this incentive scaling formally as a function within reinforcement learning that regulates the degree to which prior reward learning biases choice; that is, how much established learning is exploited. We assign this latter function to tonic dopamine and construe it as regulating *thrift*; that is, regulating the frugality of energy expenditure.

### Dopamine and goal selection: making wise choices in energy expenditure

The frugality of one's expenditures depends upon the resources available. A rich person need not quibble about thousands of dollars while a poor person needs to count pennies. Similarly, animals living in energetic environments of plenty need not worry about energy conservation while those living under conditions of scarcity must expend energy judiciously. Thus, energy management entails not only determining the overall magnitude of energy expenditure, as discussed above, but also its allocation to specific activities.

We start from the premise that when energy is readily available, energy expenditure is adaptively advantageous, for two primary reasons. First, physical activity has been shown in countless studies to contribute significantly to health and longevity (Holloszy et al., 1985; Samorajski et al., 1985; Paffenbarger et al., 1986; Holloszy, 1988; Helmrich et al., 1991; Greendale et al., 1995; Booth et al., 2000; Alevizos et al., 2005; LaMonte et al., 2005; Warburton et al., 2006; Gaesser, 2007; Huffman et al., 2008; Hawley and Holloszy, 2009; Mercken et al., 2012). Under conditions of scarcity, animals have to work hard to find food; however, under conditions of plenty they do not. A system that increased energy expenditure in response to conditions of plenty would maintain activity levels and health, at the very least precluding an animal from becoming a fat, slow morsel for a predator's dinner. Moreover, if energy is available, then there is an informational advantage to be gained from exploration that allows an animal to more fully learn about its environment, information that could be exploited in the future (Behrens et al., 2007). Thus, when energy is available, there is logic to inducing expenditure and behavioral exploration. In contrast, when energy is scarce, the animal needs to conserve its energy and maximally exploit its knowledge of the environment. In the hypothesis being developed here, dopamine's role in reward processes arises as a mechanism for the allocation of energy toward specific activities and stimuli.

To optimally allocate energy, two primary functions are required. First, the organism has to determine the value of stimuli and actions in the first place. Second, the organism needs to determine to what degree those values should be taken into account when making behavioral choices: how frugal or “value-conscious” should expenditure be? The two umbrella views of dopamine and reward, the reinforcement learning and the incentive-salience hypotheses, provide these two functions. In the sections below we examine both functions and their contribution to the explore-exploit axis (Figure 1) cast in terms of TD learning models.

### Dopamine and reinforcement learning: value accounting

As noted in the introduction, the term “reward” can be ambiguous. Here we adopt an information perspective and define reward as positively valenced outcome information. The primary question to be addressed is what role dopamine plays in linking reward history to future choices. Reinforcement learning perspectives argue that dopamine modulates corticostriatal plasticity in response to reward, i.e., positive outcome information, thus mediating learning *about* stimuli and actions that are valuable. Motivational views, specifically incentive-salience, suggest that dopamine modulates the *expression* of previously learned values (incentive). The current hypothesis encompasses both within a TD learning framework.

Temporal difference models are a class of reinforcement learning algorithms that have been successfully applied to understanding how neural substrates, such as dopamine and the basal ganglia, mediate behaviorally observed reinforcement learning. Within these models, stimuli and actions are assigned a “value” that incorporates all expected future rewards associated with those stimuli or actions. As time unfolds and the animal moves incrementally forward in time, advancing through successive states (i.e., configurations of stimuli, actions, rewards), at each step forward the prior prediction (*t* − 1) is compared to what was actually received at time *t* plus remaining expected rewards in the future, that is, the value estimate at *t*. If there is a discrepancy, called a prediction error, the prior value at *t* − 1 is adjusted so that when that state is encountered again, it will be more accurate. When the animal moves forward in time to *t* + 1, the same process will occur, this time adjusting the predicted value of *t* by comparing it to actual reward at *t* + 1 plus the future expected value of *t* + 1, and so on. The name TD arises because an entire succession of states, each with its own value prediction of “remaining” future reward, is adjusted one step at a time. It is this collection of values associated with different states—stimuli and actions—that enables accurate prediction of future reward. In short, it is an algorithm that facilitates trial and error learning where the animal is always, as it were, *in medias res*, and an accurate estimate of the value of particular stimuli and actions accrues gradually over time through experience.

Temporal difference models have two key functions. First, they learn. As described above, using an update rule based on prediction errors, they adjust prior values associated with stimuli and actions. Second, they make decisions. That is, once you have a set of values, there is a rule for how those values are used in selecting an action. These two functions are associated with two parameters, commonly known as alpha, the learning rate, and beta, the “temperature,” respectively. The learning rate determines to what degree new information alters established values, both weighting new information against old and establishing a “window of forgetting.” The temperature parameter determines the degree to which current value information (i.e., reward history) biases action selection, frequently referred to as the “explore-exploit” parameter.

Substantial evidence supports a role for dopamine in mediating reinforcement learning and corticostriatal plasticity, which will not be reviewed here (Montague et al., 1996; Schultz et al., 1997; Reynolds and Wickens, 2002; Schultz, 2002, 2010; Cannon and Palmiter, 2003; Wise, 2004; Berridge, 2007; Goto et al., 2007; Redish et al., 2007; Robbins and Roberts, 2007; Salamone, 2007; Schultz, 2007; Dayan and Niv, 2008; Kheirbek et al., 2008, 2009; Redgrave et al., 2008; Kurth-Nelson and Redish, 2009; Lovinger, 2010; Lüscher and Malenka, 2011). This learning function has been primarily associated with phasic dopamine activity operating on a millisecond timescale and is not believed to represent reward directly. Instead, drawing from TD learning as described above, phasic dopamine is believed to encode prediction errors. By signaling unanticipated reward or the failure of expected reward (Schultz et al., 1997; Schultz, 2007; Flagel et al., 2010; Brown et al., 2011; Day et al., 2011), phasic dopamine updates the values associated with stimuli and actions by altering synaptic weights through its effects on corticostriatal plasticity. Within the current hypothesis, we accept and view this function of phasic dopamine in updating value as, in a sense, an accounting function: dopamine does not set, create or arbitrarily scale value but is functionally providing a teaching signal to modify learning to accurately reflect the value associated with stimuli and actions. Put simply, this function of dopamine is trying to “get the value right”: a fundamental characteristic of any successful budget is getting the numbers right. That is, the highly detailed and mechanistic functions of dopamine in reinforcement learning enables an animal learn about its environment *in order* to better allocate and utilize its available energy.

### Dopamine and incentive-salience: energy budget allocation

The incentive-salience view of dopamine, in contrast, argues that dopamine scales the *impact* of reward-associated stimuli on behavioral choice (Cagniard et al., 2006b; Berridge, 2007). That is, dopamine modulates the degree to which incentive value associated with stimuli biases behavior. Generally, increased dopamine is viewed as increasing incentive and scaling up appetitive behavior; that is, inducing greater “wanting.” This view is consistent with the decades of literature showing increased dopamine results in increased goal pursuit and effort, as discussed above. In essence, this might be conceptualized as increased *exploitation* of reward learning: reward value exerts a greater bias on behavioral choice. However, if increasing dopamine results in increased exploitation, one might logically expect that decreasing dopamine would result in greater exploration; that is, behavior will be *less* biased by reward information. However, to our knowledge, no data have demonstrated increased exploration as a consequence of diminished dopamine. Instead, decreased dopamine has been consistently associated with decreased activity and exploratory behavior. In the behavioral flexibility home cage study described above, we found that elevated dopamine decreased coupling between reward history and choice, favoring *exploration*, not exploitation, consistent with decades of open-field studies showing increased exploratory activity as a consequence of increased dopamine (e.g., Zhuang et al., 2001). Consistent with the current hypothesis, recent computational work by Humphries and colleagues (Humphries et al., 2012) demonstrate that tonic dopamine can modulate the trade-off between exploration and exploitation. In their model, the effects of dopamine on this trade-off are complex and concentration-dependent, but indicate that high dopamine can induce exploratory behavior.

We propose that the incentive-salience view of dopamine, where dopamine scales the degree to which reward value influences behavioral choice, captures a critical effector mechanism in dopaminergic management of energy expenditure. By modulating the balance between exploration and exploitation, dopamine regulates the frugality of energy expenditure. In contrast to current theories of incentive-salience, however, we argue that dopamine acts roughly in the opposite direction from what is widely construed: dopamine favors *exploration*; that is, *diminished* biasing of behavioral choice by reward value, though as the work of Humphries et al. (Humphries et al., 2012) demonstrates, the precise operation of this function of dopamine is likely to be complex. The increased effort in reward pursuit observed by enhanced dopamine function, in this view, arises not as a consequence of enhanced reward value but as the result of a dopaminergic signal to *expend* energy and be less frugal in the pursuit of goals.

Although, through its role in reinforcement learning, (phasic) dopamine contributes to learning about value, the range of the vertical axes in Figure 1 does not represent value *per se*, from lesser to greater value, but the degree to which established values *bias* or direct behavioral choice, ranging on a continuum from heavily influencing choice (exploit) to minimal influence (explore). This could be construed as a frugality axis where greater exploitation maximizes reward for energy expended while reduced exploitation facilitates exploration and greater energy expenditure.

How and to what extent learned values direct behavioral choices clearly depends upon many factors, most obviously the motivational state of the organism, the “salience” in “incentive-salience,” taking into account the organism's internal environment. The *external* environment needs to be taken into account as well, specifically the availability of reward, particularly energy. As discussed above, the richness of the environment determines how frugal an animal needs to be with its energy expenditure and how much it needs to maximally exploit prior learning. Niv et al. (2007) have suggested that tonic dopamine encodes average reward over time, a formalization that links the relative abundance or scarcity of reward in an environment to behavioral vigor. In the model proposed by Niv, higher average reward induces greater behavioral vigor to reduce opportunity costs; that is, the more rich the environment, the more that is lost by inactivity, colorfully described as “the cost of sloth.” Focusing specifically on energy availability, we share this view that tonic dopamine signals the abundance or scarcity of energy in the environment over time. However, in the current view, rather than viewing the increased vigor associated with dopamine as inducing greater exploitation to reduce opportunity costs, we suggest that increased dopamine reflects energy abundance and induces greater energy expenditure but *less* exploitation; that is, less energy frugal behavior, favoring exploration (but not inactivity).

Finally, it is widely believed that learned values can be part of a goal-directed or a habit system (Daw et al., 2005; Balleine et al., 2007), associated frequently with the dorsomedial and dorsolateral striatum, respectively (Yin and Knowlton, 2006; Balleine and O'Doherty, 2010). In TD models, the latter is cast as a “cache” or “model-free” system where the value of stimuli and actions are inscrutable; that is, how those values were derived is not available for examination. In contrast, goal-directed behavior is associated with “model-based” systems where a “tree” of states and their associated value are explicitly represented such that the animal can deliberately search the tree to determine how value at any leaf is derived and evaluate those values against current motivational states. In contrast, the habit, or cache system is thought to be insensitive to motivational state, though importantly, not insensitive to new learning (Balleine and O'Doherty, 2010). Precisely because the cached value cannot be “examined” with regard to current goals, habitual behavior based on cached values will be emitted in response to stimuli presentation even in the absence of motivation until the cached value driving that behavior is updated. In TD models, the temperature parameter adjusts the degree to which values bias behavior without regard to the source of those values, i.e., whether they are part of a cache, model-free or model-based system. The question arises, then, as to whether the dopamine regulation of explore-exploit, suggested here, applies equally to both the habit and goal-directed system, a question we cannot answer. Insofar as reduced dopamine may induce exploitation, thrift, and conservation, increasing the control prior learning has over behavior, reward/energy poor environments and hypodopaminergia may, by increasing the influence of established learning and values on behavior, increase the control exerted by habit based systems, though this clearly requires further investigation.

### Dopamine and shifting energy expenditure: revisiting the GO and NOGO pathways

A primary target of dopamine, widely associated with dopamine's role in both reward and motor control, is the striatum (Albin et al., 1989, 1995; Alexander et al., 1990; Mink, 1996; Everitt and Robbins, 2005; Cagniard et al., 2006b; Balleine et al., 2007; Nicola, 2007; DeLong and Wichmann, 2009; Wise, 2009; Haber and Knutson, 2010; Humphries and Prescott, 2010; Sesack and Grace, 2010). The striatum is the main entry point for cortical inputs (Bolam et al., 2000) that are processed through the basal ganglia eventually returning to the cortex, comprising the well-known re-entrant corticostriatal loops (Alexander et al., 1990; Alexander and Crutcher, 1990; Alexander, 1994; Middleton and Strick, 2000; Haber, 2003; Lehéricy et al., 2005). Corticostriatal processing occurs through two parallel pathways (excluding the hyperdirect pathway), the direct and indirect that facilitate and inhibit corticostriatal throughput (Albin et al., 1995; Mink, 1996), respectively, sometimes referred to as the GO and NOGO pathways (Cohen and Frank, 2009). The GO pathway predominantly expresses D1 while the NOGO expresses D2 (Surmeier et al., 2007) such that an increase in dopamine activity enhances activity in the facilitatory GO pathway and diminishes activity in the inhibitory NOGO pathway. Conversely, reduced dopamine results in less GO activity and increased NOGO activity, an idea central to classic models of motor slowing in Parkinson's disease (Albin et al., 1989; Mink, 1996). The classic functional explanation for this dual pathway architecture is “focused selection,” the idea that the GO pathway isolates and facilitates a selected motor action while the NOGO pathway inhibits competing actions and extraneous noise, thus enabling clean execution of actions (Mink, 1996).

**Figure 2:**
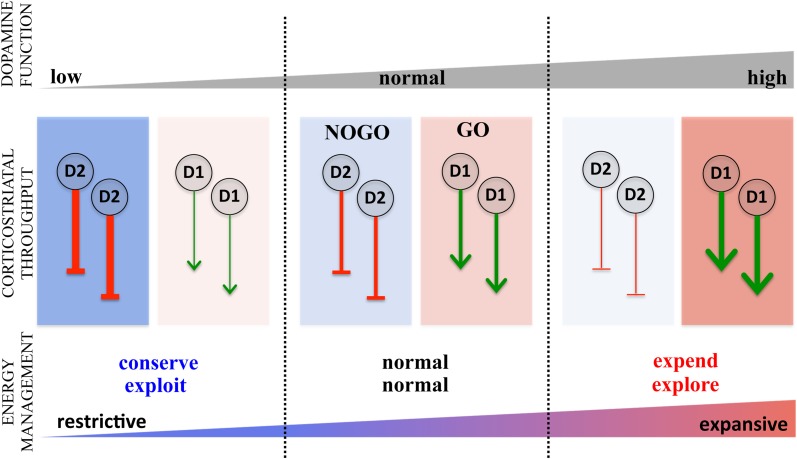
**Role of direct and indirect pathway modulation of corticostriatal throughput in regulating the energy expenditure.** The striatum modulates cortical processing via corticostriatal-thalamocortical loops through the basal gangia through two pathways, the direct, nigrostriatal (“GO”) and the indirect, striatopallidal (“NOGO”), expressing predominantly D1 and D2 dopamine receptors, respectively. Acting on D1 in the GO pathway (red toned boxes), dopamine disinhibits corticostriatal throughput facilitating activity while dopamine activation of D2 inhibits the NOGO pathway (blue toned boxes), also facilitating activity by dampening the inhibitory influence of the indirect. Conversely, decreases in dopamine diminish D1 mediated disinhibition of the GO pathway and D2 mediated inhibition of the NOGO pathway, both serving to restrict corticostriatal throughput. These dopamine effects are represented by green arrows for the GO pathways, indicating facilitation of corticostriatal throughput and red stop arrows for the NOGO pathway indicating inhibition of corticostriatal throughput. The consequences of increased and decreased dopamine on the expenditure and distribution of energy is summarized below with the two axes (conserve-expend and explore-exploit) collapses on a single scale of restrictive deployment of energy (constrained expenditure focused on exploiting reward information) versus expansive energy expenditure (high expenditure distributed liberally to behavioral activities, i.e., exploration), where restrictive energy use is represented by blue and expansive energy use by red.

This same architecture, however, can be construed as an effector mechanism for regulating energy expenditure. By shifting the balance between the GO and NOGO pathways, dopamine regulates both the total throughput of the corticostriatal system as well as modulating its selectivity (Beeler, 2011). Specifically, increased dopamine favors the GO pathway and diminishes the NOGO pathway. This results in greater overall corticostriatal throughput and greater exploration as the potential actions represented in corticostriatal input to the GO pathway face less opposition in the inhibitory NOGO pathway, reducing constraints on selecting actions. In contrast, when dopamine decreases, there is greater inhibitory, NOGO activity resulting in overall less corticostriatal throughput and greater exploitation as the actions selected through the GO pathway must be sufficiently strong to overcome the inhibitory influence of the NOGO pathway (for a learning perspective on this, see Frank et al., 2009). This provides a basis for understanding how elevated dopamine can *increase* activity overall as well as *decrease* the selectivity of that activity, i.e., increase energy expenditure and exploration. In contrast, reduced dopamine would decrease overall activity but increase selectivity, resulting in decreased energy expenditure and increased *exploitation* (Figure 2). A more detailed discussion of the role(s) of dopamine in modulating corticostriatal throughput can be found elsewhere (Beeler, 2011). This dual pathway architecture, then, provides a potential basis for modulating energy expenditure along the two axes described: regulating overall, generalized activity (conserve-expend) on one hand and, on the other hand, regulating the degree to which prior learning about reward is exploited, the explore-exploit axes.

### Dopamine and its diverse targets: orchestration or evolutionary bric-a-brac?

In the previous section, we focus on dopamine actions in the striatum, widely construed as a primary substrate for reinforcement learning and implicated in both motivation and motor control. Dopamine, however, projects broadly across the brain, with important projections, for example, to the prefrontal cortex. Moreover, aside from the widely studied nigrostriatal and mesoaccumbens pathways, projecting to the dorsal and ventral striatum and associated with motor control and habit, and motivation, respectively, there are much less studied dopamine nuclei, including those associated with the hypothalamus, all of which may (or may not) contribute to energy management in potentially different ways. The development of a dopamine and energy management hypothesis demands asking how dopamine modulation in different targets may contribute to coordinated energy management. At this point, we cannot reasonably speculate on this question.

Reflecting on this question, however, provokes the question of whether there really is some overriding “function” of dopamine at all? Or has dopamine acquired various, unrelated functions so that the notion of a finding “the” function of dopamine represents a fool's errand: perhaps the dopamine functions we observe today evolved as patchwork, evolutionary bric-a-brac, an opportunistic collection of fortuitous adaptations. From the literature, one gets the sense that many believe there *is* an overall, grand function of dopamine—most frequently, “reward” processing, in some form or another. It seems not unreasonable to think that as the function of a neurotransmitter elaborates and transforms over evolution, those elaborations arise as variations on a theme, a further enhancement of some function that has already conferred adaptive fitness. Even if such a notion ultimately proves false, seeking a “common thread” among seemingly diverse, unrelated functions may provide deeper insight into how these apparently disparate functions interact to contribute to behavioral adaptation. “Mediating reward,” as broad and ambiguous as it is, has served as this common denominator in much of the literature and has yielded rich results. Here we suggest an alternative, overarching function, energy management.

Returning to the question of how dopamine action at different neural substrates might contribute to energy management, we can only reflect back the question from the perspective of our hypothesis. Energy management constitutes a fundamental biological process, much like reproduction, growth and responding to stress, and requires *coordinated* activity across multiple substrates in response to external conditions, in a way that reward, an event in the external world, perhaps does not. This coordinated orchestration of multiple substrates toward a single goal or purpose is characteristic of hormones, such as reproductive or stress hormones. Given dopamine's diffuse projections and actions on multiple targets, it is tempting to entertain the notion that dopamine may have evolved a hormone-like function to coordinate multiple neural substrates and orchestrate behavior optimally adapted to the prevailing energy environment in which the organism finds itself. Though admittedly broad and speculative (but see Ugrumov et al., 2012), it is interesting to ask, then, how dopamine modulation in the dorsal versus ventral striatum, or in the prefrontal cortex versus the hypothalamus, might represent the coordination of diverse neural substrates with disparate functions toward a single purpose, adapting behavior to the energy environment in which the organism must work out its survival.

## Dopamine and obesity: an alternative perspective

In recent years there has been an increased focus on the role of dopamine in obesity with several high profile papers likening overeating to a food addiction (Volkow and Wise, 2005; Trinko et al., 2007; Avena et al., 2008; Corwin and Grigson, 2009; Dagher, 2009; Davis and Carter, 2009; Ifland et al., 2009; Pelchat, 2009; Johnson and Kenny, 2010; Volkow et al., 2010). These theories focus on the role of dopamine in mediating reward processes, suggesting that in modern western cultures in which highly palatable foods are readily available, these tasty foods act similarly to drugs of abuse and induce increased “wanting” that leads to a loss of control over consumption, despite, like an addict, individual intentions to curb caloric intake (Berridge et al., 2010; Berthoud et al., 2011). There are two opposite takes on this (Davis et al., 2007; Davis and Carter, 2009). In the first, *increased* dopaminergic function arising from escalating reinforcement associated with palatable food leads to increased motivation that overrides normal homeostatic control (Finlayson et al., 2007; Zheng et al., 2009; Avena and Bocarsly, 2011). In essence, we lose control in the face of really tasty food, an effect believed to be mediated by the persistent over-activation of dopamine. In contrast, a “reward-deficiency” hypothesis has been proposed in which a *deficit* in dopamine function results in diminished reward signaling which generates excessive consumption as the person or animal tries to “fill the reward void” (Wang et al., 2004; Geiger et al., 2009; Kenny, 2010).

Within the current hypothesis, we posit that increased dopamine facilitates greater energy expenditure and exploration, i.e., *less* biasing of behavioral choice by reward, favoring less energy storage and providing protection from obesity, consistent with the well-known effects of psychostimulants in inducing weight loss (Vanina et al., 2002; Leddy et al., 2004). In contrast, decreased dopamine induces energy conservation and exploitation i.e., greater biasing of behavior by reward. In this case, energy consumption and storage is favored resulting in overeating and weight gain. Notably, in contrast to psychostimulants, anti-psychotics that antagonize dopamine (primarily D2) have been consistently associated with weight gain for decades (Allison and Casey, 2001; Vanina et al., 2002), though the exact mechanisms remain uncertain.

### Leptin, insulin, and dopamine: reconciling resources and expenditures

Observations of reduced dopamine function in obesity, particularly reduced expression of the D2 receptors, has generated the reward deficiency hypothesis (Blum et al., 2000, 2011) in which over-consumption is driven by an attempt to compensate for reduced reward signaling. In the hypothesis proposed here, we would reinterpret these data. When dopamine function is reduced, this favors energy conservation and exploitation of reward information: that is, “consume and move as little as possible,” an obvious recipe for obesity.

#### Motivational dissociation: needing without “wanting”

Decades of elegant work have demonstrated beyond question that circulating hormones that signal energy supplies, particularly insulin and leptin, contribute to the regulation of consumption and body weight through actions on the central nervous system [see Figlewicz and Sipols, 2010 for excellent review]. Consistent with its role in mediating homeostatic energy balance, leptin, and insulin act on multiple targets in the hypothalamus involved in the regulation of feeding, including NPY, POMC, α-MSH, and AgRP (Figlewicz and Sipols, 2010). These observations are consistent with drive reduction theories of motivation where energy deficit or surplus promotes or inhibits consumption, respectively.

The dramatic increase in obesity in recent years (Ford and Mokdad, 2008), however, raises the question as to why these homeostatic mechanisms apparently fail. It is widely believed that midbrain dopamine systems that mediate incentive motivation—motivation that arises from the reward value associated with stimuli (including food) rather than from need state— mediates this homeostatic failure by promoting so-called non-homeostatic or hedonic feeding (Saper et al., 2002; Zheng and Berthoud, 2007; Lutter and Nestler, 2009; Zheng et al., 2009; Berthoud et al., 2011). Considerable evidence suggests that both leptin and insulin can modulate midbrain dopamine function (Krügel et al., 2003; Fulton et al., 2006; Hommel et al., 2006; Roseberry et al., 2007; Leinninger et al., 2009) and alter food pursuit and consumption (Sipols et al., 2000; Figlewicz et al., 2001, 2004, 2008, 2006; Hommel et al., 2006; Morton et al., 2009; Davis et al., 2010b).

In the prevailing view, a great deal of data appears to suggest that both insulin and leptin decrease dopamine function, decreasing, in turn, incentive driven pursuit and consumption of food. In essence, leptin and insulin, by signaling adequate energy, represent a functional satiety signal diminishing the reward associated with food (Morton et al., 2009; Davis et al., 2010b; Figlewicz and Sipols, 2010; Opland et al., 2010; Vucetic and Reyes, 2010). Conversely, when energy is low, leptin, and insulin decline, disinhibiting dopamine and promoting enhanced incentive/reward driven food-seeking. This general idea is consistent with data demonstrating that increasing leptin and insulin reduces reward-driven behavior (Carr et al., 2000; Fulton et al., 2000; Sipols et al., 2000; Figlewicz et al., 2004, 2006, 2007; Hommel et al., 2006; Farooqi et al., 2007; Rosenbaum et al., 2008; Morton et al., 2009) and, conversely, that food-restriction, associated with decreased leptin/insulin (Havel, 2000), increases reward-driven behavior (Carroll and Meisch, 1980; Carr, 2007, 2011; Davis et al., 2010a). In short, by up and down regulating dopamine function, leptin and insulin modulate the incentive associated with food and quite possibly reward sensitivity generally (Morton et al., 2006; Davis et al., 2010a). Though this account of leptin and dopamine is intuitive, the story may nonetheless be more complex (Palmiter, 2007).

In the context of obesity, the relationship between leptin/insulin, dopamine and reward behavior is paradoxical and doesn't conform to the idea just outlined. First, rather than the expected increase in leptin/insulin signaling commensurate with increased caloric intake and adiposity, obesity has been associated with reduced sensitivity to leptin/insulin (Arase et al., 1988; Lin et al., 2001; Wang et al., 2001b; Myers, 2004; Figlewicz et al., 2006; Enriori et al., 2007; Davis et al., 2010a; Figlewicz and Sipols, 2010; Koek et al., 2012). Second, while we might expect this observed reduction in leptin/insulin sensitivity to result in increased dopamine function, analogous to reduced leptin/insulin signals in food-restriction, most studies report *decreased* dopamine function in obesity (Di Chiara et al., 1998; Wang et al., 2001a; Davis et al., 2008; Geiger et al., 2008, 2009; Li et al., 2009; Vucetic and Reyes, 2010). Finally, one might expect reduced dopaminergic function to result in decreased consumption, as the evidence cited above suggest. Instead, reduced dopamine and hyperphagia co-occur. In obesity, then, the leptin/insulin -> dopamine -> reward chain is inverted at each step.

The first two inversions highlight a critical distinction between acute and chronic positive energy balance. Reduced sensitivity to leptin/insulin is widely believed to arise as a consequence of *chronic* positive energy balance, leading to obesity and metabolic disorders, and representing a pathological adaptation. The paradoxical reduction of dopamine function associated with obesity despite reduced sensitivity to leptin/insulin is likely a (pathological) adaptation as well, as frequently proposed in theories likening obesity to addiction (Volkow and Wise, 2005; Trinko et al., 2007; Avena et al., 2009; Lutter and Nestler, 2009; Avena and Bocarsly, 2011). This severely complicates investigation because it means that for every observation of a relationship between energy intake, leptin/insulin and dopamine we have to ask “does this observation reflect normal function or pathological adaptation?” This situation increases the risk of making inappropriate inferences of normal function from pathological conditions and vice-versa, an issue addressed below (cage-induced obesity section).

The third inversion—that decreased dopamine function associated with obesity promotes rather than inhibits consumption—superficially contradicts the entire notion that dopamine enhances incentive value. However, this may reflect the complexity of neural substrates controlling consumption. In particular, reduced incentive motivation and hyperphagia can coexist. Davis et al. (Davis et al., 2010b) recently provide data suggesting that leptin modulates dopamine through two mechanisms: direct signaling through leptin receptors on midbrain dopamine cells and indirectly through leptin expressing neuron in the lateral hypothalamus to modulate dopamine cell activity. They suggest that leptin's actions on the LH regulate homeostatic motivation while its actions on midbrain dopamine regulate effortful responding. Though these two mechanisms normally work in concert, they can become dissociated such that consumption and willingness to work for food are not correlated (e.g., Greenwood et al., 1974; Salamone et al., 1991; Baldo et al., 2002; Davis et al., 2010b; Rasmussen et al., 2010). If non-homeostatic feeding is effort to obtain food—“wanting”—in the absence of need (Berridge et al., 2010), the pathological adaptation of leptin/insulin/dopamine to obesity may reflect the opposite: a perceived need to consume food without expending effort, needing without “wanting.”

#### Dopamine and energy homeostasis: an expenditure-centric perspective

This work described above focuses almost exclusively on consumption, the intake side of energy balance. Leptin, though less systematically investigated, also plays a role in regulating energy expenditure (Pelleymounter et al., 1995; Williams et al., 2001; Elmquist et al., 2005; Ludwig et al., 2005; van de Wall et al., 2008; Leinninger et al., 2011; Ribeiro et al., 2011). However, despite dopamine's well-known role in regulating activity, little is known about how the interactions between leptin, insulin and dopamine modulate activity and energy expenditure. From the perspective of energy expenditure, one might expect that leptin/insulin, signaling energy availability, would enhance energy expenditure and increase activity (Ribeiro et al., 2011)—“if you got it, use it”—which is inconsistent with observations that leptin decreases dopamine function. However, recent work (Leinninger et al., 2009; Opland et al., 2010) suggests the relationship between leptin and dopamine may not be simple and unidirectional. Leinninger and colleagues suggest that leptin acting on the LH *increases* dopamine function (Leinninger et al., 2009) while activation of leptin receptors on dopamine cells decreases dopamine function (Hommel et al., 2006; Figlewicz and Benoit, 2009). Leshan et al. (2010) suggest that the dopamine cells that express leptin receptors represents a small (~10%) subpopulation that projects almost exclusively to the central nucleus of the amygdala. When leptin increases from a surplus of energy, then, its primary effect on dopamine, via LH, may be to *increase* dopamine function and enhance activity and energy expenditure, as observed by Ribeiro and colleauges (Ribeiro et al., 2011): energy is available, use it. The dopamine cells and projection that directly express leptin receptors may play a different role in appetitive *learning*— associated with the CeN (Holland and Gallagher, 1993; Parkinson et al., 2000; Connor et al., 2001; Baxter and Murray, 2002; Lee et al., 2005; Paton et al., 2006; El-Amamy and Holland, 2007)—an intriguing idea beyond the scope of the current discussion. In the hypothesis proposed here, this leptin-mediated increase in dopamine does not enhance reward, but rather shifts regulation of activity toward greater expenditure and greater exploration. Greater exploration results in an apparent reduction in reward sensitivity/incentive as behavior is *less biased* by reward, though importantly, still motivated.

The reward centric view of dopamine places the up- or down-regulation of reward and incentive motivation as dopamine's primary contribution to energy homeostasis and obesity. The current hypothesis centers the role of dopamine on energy expenditure and suggests that available energy normally increases dopamine resulting in increased activity and exploration, where the impact of reward on behavioral choice is actually diminished. Conversely, low energy would decrease dopamine, resulting in energy conservation and exploitation of reward information that is, *increasing* the impact of reward on behavior. The latter is consistent with observations often cited to support the “reward deficiency” hypothesis but here we interpret these data as reflecting a “reward exploit excess.” This hypothesis would suggest that high caloric intake should increase dopamine and, through increased activity, be protective against obesity. This putative mechanism, however, does not appear any more successful than homeostatic mechanisms in preventing obesity in our current environment. Why?

### Thwarting energy expenditure: cubicle and cage induced obesity

This hypothesis would predict that a ready supply of energy, as is generally the case in modern western societies, would enhance dopamine and facilitate energy expenditure, providing *protection* against obesity. Critically, this effect is contingent upon the *opportunity* to expend energy. Dietary induced obesity (DIO) in rodents fed high fat, high calorie diets is a prevalent model of environmentally induced obesity. Though widely construed as a model of obesogenic environments, rodents on such diets do not universally become obese, showing degrees of resistance that vary between strains (Brownlow et al., 1996; Funkat et al., 2004; Novak et al., 2010) and between individuals, the basis for selective breeding of obesity susceptible and resistant rodents (Levin, 2010). Assessing the role of dopamine-mediated effects on energy expenditure in DIO is difficult as the question has not been investigated directly.

Although wheel running has been shown to protect against DIO in several models of obesity (Zachwieja et al., 1997; Levin and Dunn-Meynell, 2004; Bi, 2005; Moran and Bi, 2006b; Patterson et al., 2008, 2009; Meek et al., 2010; Novak et al., 2010), the extent to which such protective voluntary activity is modulated by dopamine has not been directly examined in these studies. Obesity-prone OLEF rats show greatly decreased obesity when provided running wheels (Bi, 2005). Interestingly, the meal patterning of these rats, compared to controls, is analogous to what we observe with the DAT mice: they consume larger but fewer meals, though unlike the DAT their net consumption is elevated (Moran and Bi, 2006a, p.1214, Figure 2). Younger, non-diabetic OLETF rats show increased extracellular dopamine, consistent with the DAT meal patterning phenotype (Anderzhanova et al., 2007). However, at more advanced pre-diabetic and diabetic ages, they show a dramatic decline in dopamine (Anderzhanova et al., 2007). One interpretation of these data is that elevated dopamine levels in these rats predisposes them to greater energy intake *and* expenditure but in the absence of voluntary exercise opportunities, the increased energy expenditure is blocked, resulting in energy imbalance, obesity, and metabolic disorder.

Some studies have demonstrated that high fat/calorie diets diminish dopamine function, including reduced TH, reduced stimulated dopamine release and reduced D2 receptor expression (Geiger et al., 2008, 2009). However, whether the observed reduction in dopamine function arises as a direct consequence of the increased availability of energy or secondary to other pathophysiology is unclear. Specifically, high fat/calorie diets have been associated with leptin and/or insulin insensitivity (Arase et al., 1988; Lin et al., 2001; Wang et al., 2001b; Myers, 2004; Figlewicz et al., 2006; Davis et al., 2010a; Figlewicz and Sipols, 2010). This makes interpreting alterations in dopamine function in response to DIO difficult. For example, though reduced D2 function has been reported with DIO, these observations generally occur after many weeks of high fat diet, introducing the possibility that these changes arise secondary to chronically elevated leptin and insulin and subsequent leptin/insulin insensitivity. Unfortunately, this possibility is rarely addressed and insulin/leptin levels are not typically reported. However, in one study that examined D2 and DAT expression after 20 days of HF diet and reported insulin/leptin levels, the authors observed a decrease in DAT and an *increase* in D2 (South and Huang, 2007; see also Huang et al., 2005), both consistent with increased activity. In a more recent study, these same authors observe an increase in dopamine system function when rats are switched from chow to high energy diet (South et al., 2012). These data suggest that the *initial* response to high fat and increased caloric availability is to *increase* dopamine and *increase* expression of the disinhibitory D2 receptor, inducing greater activity. In order to answer the question of how the dopamine system responds to an abundance of available calories, it is critical to disambiguate *initial* from *chronic* response and assess the degree to which pathological adaptations, such as leptin insensitivity, are present.

To what degree do the changes observed in the dopamine system under DIO arise not from high calorie diet itself but from a lack of exercise opportunity? That is, in observations of diminished dopamine function associated with DIO, we can ask not only to what extent does this reflect a pathological adaptation, but also to what degree does a lack of opportunity for voluntary energy expenditure *contribute* to this pathology? Evidence suggest that wheel running can alter functional characteristics of the dopamine system (MacRae et al., 1987; Sabol et al., 1990; Hattori et al., 1993, 1994; Wilson and Marsden, 1995; Liste et al., 1997; Meeusen et al., 1997; Foley and Fleshner, 2008; Greenwood et al., 2011), including increased extracellular dopamine, increased turnover, elevated TH mRNA and changes in D2 expression. In one recent study that differentiated between postsynaptic D2 and autoreceptors, the autoreceptors were found to be downregulated and the postsynaptic D2 upregulated (Foley and Fleshner, 2008). Moreover, voluntary activity may mitigate pathological adaptations in leptin and insulin signaling that may, indirectly, protect dopamine function (Krawczewski Carhuatanta et al., 2011). Thus, voluntary exercise may ameliorate the diminished dopamine function observed in DIO, though this has not been systematically examined.

In the hypothesis proposed here, increased availability of energy would, via up-regulation of dopamine mediated behavioral activity, result in increased exploration and energy expenditure, allowing the animal to take advantage of a plentiful energy supply and protect against obesity. Insofar as the DIO paradigm does not provide an opportunity for exploration and energy expenditure by confining rodents to small cages with little to no novelty, stimulation or exercise opportunities, it may reflect the consequences of *thwarting* energy expenditure under conditions of energetic abundance. Many have suggested that sedentary lifestyles characteristic of modern western cultures may contribute to obesity as much or more than diet (Powell and Blair, 1994; Booth et al., 2000; Hill et al., 2003; Chakravarthy and Booth, 2004; Levin and Dunn-Meynell, 2004; Warburton et al., 2006; Booth and Lees, 2007; Elder and Roberts, 2007; Hawley and Holloszy, 2009; Chaput et al., 2011), making DIO highly relevant to understanding obesity in modern societies. However, whether the induced obesity arises from increased caloric consumption or from a lack of meaningful opportunities to expend energy—cage- or “cubicle”-induced obesity—remains unclear. Equally, though widely proposed that dopamine contributes to DIO by modulating reward and appetitive motivation, its potential contribution through modulating energy expenditure remains largely uninvestigated and, in the conventional DIO paradigm, obscured.

## Future directions: reformulating research strategies

The reward hypothesis of dopamine has dominated investigation and thinking about dopamine; experiments are typically designed within that conceptual framework. As a consequence, the information necessary to evaluate the proposed hypothesis is more often than not missing. Frequently, activity levels are simply ignored. This ranges from broad, overall investigation in which there is a profound bias toward studying appetitive behavior and consumption with only recently a nascent literature on mechanisms controlling voluntary energy expenditure to specific experiments, such as many DIO studies in which activity is not measured or taken into account at all [e.g., Geiger et al., 2008, 2009]. This bias toward reward theories is also reflected in the literature in which elaborate and sophisticated pathways controlling ingestion and/or reward have been extensively mapped out while a comparable mapping of mechanisms and pathways regulating voluntary activity is virtually non-existent (but see Garland et al., 2011). Integrating systematic and agreed upon measures of activity needs to become routinely integrated into studies of dopamine function.

Second, adaptation is environment-dependent. Currently, the vast majority of animal studies effectively investigate one energy economy: periodic, temporary food scarcity arising from food restriction employed to motivate animals. Not only does this fail to reflect the *range* of conditions to which an animal must adapt, but it does not reflect the primary condition believed to underlie the rise in obesity, which is an environment of plenty, not scarcity. A semi-naturalistic home cage approach, as illustrated by our work as well as others (Hursh et al., 1988; Chaney and Rowland, 2008) is essential to obtaining a “complete” picture of dopamine function. In such home cage paradigms, the environmental contingencies can be controlled along different dimensions over a sustained period without the need for artificially induced energy deficits (i.e., food restriction) or the introduction of artificial temporal horizons (the hour session); that is, the animal's self-regulated behavior in response to its environment can be more fully investigated.

Third, virtually all work focuses on the impact of altered dopamine on behavior with little examination of how behavioral interactions with the environment and subsequent outcomes alter the dopamine system itself. These types of studies are admittedly challenging, as evidenced in the literature on how stress alters dopamine function. Nonetheless, they seem critical to fully understanding the adaptive function of dopamine. Does a sustained environment of plenty or scarcity up- or down-regulate dopamine function? Though the question may well be critical, there is no clear or compelling answer to date and the question is rarely asked.

## Conclusions: a broader view

Here we develop a broad hypothesis of dopamine function which suggests that the myriad apparent functions of dopamine might be understood collectively as mechanisms by which energy expenditure is adapted to the energy economy in which the animal finds itself: a substrate for reconciling the pursuit of reward with resources. We first elaborated this hypothesis in theoretical terms attempting to integrate different views of dopamine function into a broader energy management framework. We then applied this framework to re-interpreting ideas and data in the growing field of dopamine and obesity. We propose the novel hypothesis that dopamine, by favoring energy expenditure, would normally be protective against obesity but that the sedentary lifestyles of modern society impede this protective process and induce pathological adaptations that contribute to rather than protect against obesity. Though beyond the scope of the current discussion, we believe the framework sketched here in broad strokes may be fruitfully applied to other areas of research in dopamine, including attention-deficit hyperactivity disorder and addiction, potentially yielding novel insights and testable hypotheses.

Reward—the linkage of external events and stimuli to internal needs—clearly represents a critical function from an evolutionary, adaptive point of view. However, reward and value are fundamentally relative. What is the yardstick by which reward is modulated? Here we suggest that the dopamine system, at its root, arose to address an even more fundamental function than reward: optimally utilizing energy resources, the very heart of adaptive survival and, literally, a matter of life or death.

### Conflict of interest statement

The authors declare that the research was conducted in the absence of any commercial or financial relationships that could be construed as a potential conflict of interest.
